# Hepatitis B Virus Reactivation in Cancer Patients Undergoing Immune Checkpoint Inhibitors Therapy: A Systematic Review

**DOI:** 10.7150/jca.77247

**Published:** 2022-10-31

**Authors:** Jian Zhao, Yuehua Zhang, Siyuan Qin, Bingwen Zou, Yongsheng Wang

**Affiliations:** 1 Clinical Trial Center, National Medical Products Administration Key Laboratory for Clinical Research and Evaluation of Innovative Drugs, West China Hospital, Sichuan University, Chengdu, Sichuan, 610041 P.R. China.; 2 West China School of Public Health and West China Fourth Hospital, Sichuan University, Chengdu, Sichuan 610041, P.R. China.; 3 State Key Laboratory of Biotherapy and Cancer Center, West China Hospital, and West China School of Basic Medical Sciences & Forensic Medicine, Sichuan University, and Collaborative Innovation Center for Biotherapy, Chengdu, 610041, P.R. China; 4 Department of Thoracic Oncology, Cancer Center and State Key Laboratory of Biotherapy, West China Hospital, Sichuan University, Chengdu, China.

**Keywords:** immune checkpoint inhibitors (ICIs), immunotherapy, hepatitis B virus (HBV), reactivation, Food and Drug Administration Adverse Event Reporting System (FAERS)

## Abstract

**Background:** Immune checkpoint inhibitor (ICI) therapy is now administered to patients with advanced cancers. However, the safety and efficacy of ICIs in cancer patients with hepatitis B virus (HBV) infection is unknown. Therefore, we performed this systematic review to examine the safety and efficacy of ICIs in patients with HBV infection, with particular focus on HBV reactivation.

**Methods:** Studies examining ICI treatment in patients with advanced cancer and HBV infection in PubMed from database inception to April 2022 were retrieved in accordance with the Preferred Reporting Items for Systematic Reviews and Meta-Analyses guidelines. In addition, reports of individuals diagnosed with HBV reactivation were supplemented through the Food and Drug Administration Adverse Event Reporting System.

**Results:** We identified 20 articles (8 case reports, 10 retrospective case series, and 2 prospective clinical trials) and 2 meeting abstracts including 633 patients with advanced cancer and HBV infection treated with ICIs. The overall rate of HBV reactivation was 4.1% (26/633), and no HBV-related fatal events were reported. Among patients with HBV reactivation with known baseline data (20/26), HBV-DNA returned to undetectable status in 15 of 17 patients (88.2%) after a median 5.5 weeks (range, 1-14 weeks). Therapeutic responses to ICIs were observed in 14 of 88 patients (15.91%) with hepatocellular carcinoma, 6 of 45 patients (13.33%) with non-small cell lung cancer, and 3 of 13 patients (23.08%) with melanoma.

**Conclusion:** ICIs may be safe and effective in patients with advanced cancer and HBV infection. However, there is still a need for clinical monitoring of liver enzymes and HBV-DNA during ICI therapy. Prospective trials are necessary to elucidate the appropriate antiviral therapy in these patients.

## Introduction

Immune checkpoint inhibitors (ICIs) targeting programmed cell death 1 (PD-1), programmed cell death ligand 1 (PD-L1), and cytotoxic T lymphocyte antigen 4 (CTLA-4) have become a well-established therapeutic option for cancer 1.

The World Health Organization estimates that there were approximately 296 million chronic hepatitis B virus (HBV) infections worldwide in 2019 2. A large proportion of patients with cancer may also have HBV infections. A multicentre prospective study reported that 0.6% of patients with newly diagnosed cancer had chronic HBV infections, whereas 6.5% had previously had HBV infections 3. However, clinical trials of immunotherapy for cancer exclude patients with HBV infection, and few prospective clinical studies include individuals with chronic HBV infection 4,5. Therefore, the safety and efficacy of ICIs in cancer patients with HBV are unclear.

Basic studies have shown that chronic HBV infection leads to virus-specific T cell exhaustion 6,7, which could theoretically affect the efficacy of ICIs. Furthermore, reactivation of HBV during treatment can result in treatment delay or termination. However, whether ICIs increase the risk of HBV reactivation and whether there is ongoing need for antiviral prophylaxis are unknown. Therefore, we performed a systematic review of the current literature to provide evidence for clinical decision making.

## Methods

### Data source and search strategy

This systematic review follows the Preferred Reporting Items for Systematic Reviews and Meta-Analyses guidelines. We comprehensively searched the PubMed, Medline and Embase databases for studies examining ICIs and HBV infection from database inception to April 2022 using the following terms: ("Hepatitis B virus*" OR "HBV") AND ("exacerbation" OR "“reactivation" OR "flare" OR "recurrence") AND ("PD-1" OR "PD-L1" OR "CTLA-4" OR "Immune checkpoint inhibitor" OR "ipilimumab" OR "nivolumab" OR "pembrolizumab" OR "atezolizumab" OR "durvalumab" OR "avelumab" OR "tremelimumab" OR "toripalimab" OR "sintilimab" OR "cemiplimab" OR "camrelizumab" OR "tislelizumab"). We obtained 400 articles. In addition, three meeting abstracts were identified after reviewing annual meeting proceedings from the American Society of Clinical Oncology (ASCO), Society of Immunotherapy of Cancer, American Association for Cancer Research, American Society of Hematology, and American Association for the Study of Liver Diseases 8-10. All publications were screened by two investigators independently.

### Inclusion and exclusion criteria

**Figure 1:**
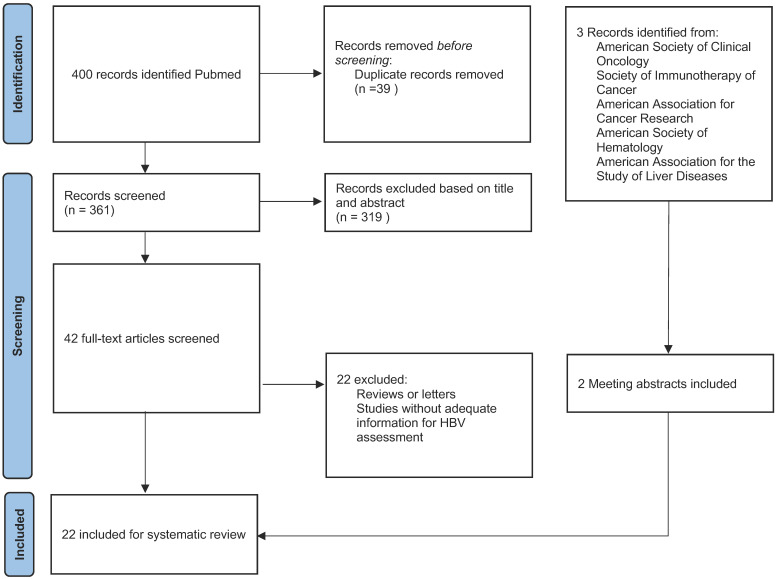
PRISMA Diagram Detailing Article Selection

The inclusion criteria were: (1) Patients with malignant tumors. (2) Patients treated with anti-PD-1/ PD-L1 or CTLA-4 monotherapy or in combination with other anti-tumour therapy. Studies were excluded if they were: (1) Reviews or letters. (2) Studies focused on the mechanism of HBV reactivation. (3) Studies without adequate information on HBV reactivation. Eventually, 22 publications were included** (Fig. 1)**. We also screened related review articles to ensure comprehensive retrieval 1,11-15.

**Figure 2:**
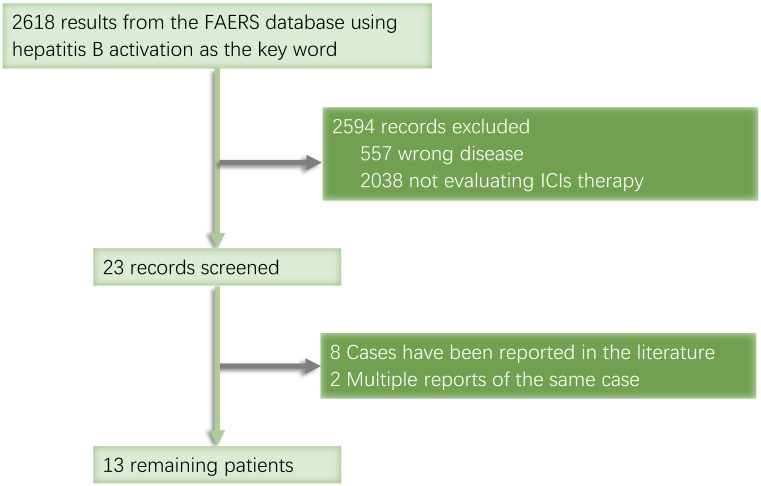
FAERS database retrieval flowchart

When searching the ASCO website, we found a conference report titled 'Hepatitis B reactivation with pembrolizumab, atezolizumab, and nivolumab: A pharmacovigilance study and literature review' in the Food and Drug Administration Adverse Event Reporting System (FAERS). Although the full text was not available, we used this open database to search for more records on HBV reactivation, from which we gathered a total of 2,618 records collected between January 2013 and September 2020. We identified 12 cancer patients treated with ICIs whose cases were not duplicated or published **(Fig. 2).**

### Statistical analysis

We used descriptive statistics to summarize the study findings. Statistical calculations were performed with Excel (Microsoft Excel, Microsoft Office, Redmond, Washington).

## Results

We included 633 patients with advanced cancer and HBV infection treated with ICIs in 22 studies (8 case reports 16-23, 10 retrospective case series 24-33, 2 prospective clinical trials, and 2 meeting abstracts 8,9) (**Table 1**). Since one study included patients with HBV and hepatitis C virus infections (n = 16) in a composite cohort and two meeting abstracts lacked patient data (n = 79), 95 patients were excluded. The baseline characteristics of 538 patients are summarized in **Table 2**. Among these eligible patients, the most prevalent cancer type was hepatocellular carcinoma (HCC) (353/538 [65.6%]), followed by non-small cell lung cancer (NSCLC) (70/538 [13.0%]), and melanoma (46/538 [8.6%]). A total of 416 patients (77.3%) received antiviral prophylaxis, of whom 57.7% received entecavir, 15.6% received tenofovir, and 3.1% received lamivudine; data on the antiviral prophylaxis regimen was not available for 96 patients (23.1%).

The FAERS searches yielded 2,618 potentially relevant patients, of whom 12 had cancer and developed HBV reactivation during ICI treatment (**Table 3**). The first HBV reactivation was reported in 2015. The main cancer types were lung cancer (n = 3) and non-Hodgkin lymphoma (n = 2). With respect to specific medications, six patients were administrated pembrolizumab, three were administered nivolumab, two were administered atezolizumab, and one was administered durvalumab. Four patients received ICIs in combination with other drugs, including two with non-Hodgkin lymphoma who were treated with rituximab and brentuximab vedotin, which respectively target CD20 and CD30, one with NSCLC who was treated with glucocorticoids and pembrolizumab, and one with triple-negative breast cancer who received paclitaxel with atezolizumab. Four of these patients died, but their causes of death were not described in detail.

### Safety

The main adverse events related to laboratory test results were elevated liver aminotransferase, including 18 cases of Common Terminology Criteria for Adverse Events (CTCAE) grade 3-4 aspartate aminotransferase (AST) elevation and 36 cases of CTCAE grade 3-4 alanine aminotransferase (ALT) elevation. Other severe adverse events (CTCAE grade 3 or 4) included hyperglycaemia (n = 3), thrombocytopenia (n = 1), and neutropenia (n = 1). Treatment-related CTCAE grade 3-4 adverse events included diarrhoea (n = 1), pneumonia (n = 1), and colitis (n = 1) **(Table 4).**

HBV reactivation developed in 26 patients (4.1%), with a median onset of 11 weeks (range, 2-35 weeks) after ICI therapy, 6 of whom were excluded from our analysis because of missing baseline data (**Table 5**) 26. The tumour types of the remaining 20 patients were HCC (n = 8), NSCLC (n = 5), nasopharyngeal carcinoma (n = 2), melanoma (n = 2), metastatic adenocarcinoma of the lung (n = 1), head and neck squamous cell cancer (n = 1), and soft tissue sarcoma (n = 1). Seven of these patients were treated with pembrolizumab, five with nivolumab, four with toripalimab, two with camrelizumab, and one with sintilimab. Notably, HBV reactivation occurred in one patient who underwent four cycles of ipilimumab and one follow-up cycle of nivolumab. Thirteen of these patients received ICI monotherapy, six received ICIs combined with hepatic arterial infusion chemotherapy (HAIC), and one received ICIs with whole brain radiotherapy.

Only five patients had a detectable HBV-DNA viral load before ICI treatment. At reactivation, the median HBV-DNA viral load was 3.92 × 10^3^ copies/mL. Fifteen patients experienced hepatitis, with a median peak ALT (n = 15) of 281 U/L (range, 151-994 U/L) and a median peak AST (n = 5) of 520 U/L (range, CTCAE G1 to 845 U/L).

Eleven patients were treated with prophylactic antivirals (eight received entecavir and three received tenofovir) before ICI therapy. Two patients diagnosed with immune-related hepatitis received glucocorticoids before being diagnosed with HBV-related hepatitis 18,29. After HBV reactivation, 11 patients were treated with entecavir, 6 with tenofovir, and 1 with entecavir combined with tenofovir; the type of treatment was not noted for 2 patients. HBV-DNA returned to undetectable status in 15 of 17 patients (88.2%) after a median 5.5 weeks (range, 1-14 weeks). One patient (**Table 5,** patient No. 3) had undetectable HBV-DNA before immunotherapy. After 28 weeks of pembrolizumab treatment, there was an increase in HBV-DNA but no increase in AST or ALT. However, the patient's HBV-DNA returned to undetectable status after 5 weeks without antiviral therapy 31. One patient with both HIV and HBV infections before administration of ICIs, received anti-HIV treatment with dolutegravir and abacavir, and the HIV-RNA copies appeared to be normal (<20 copies/mL). Therefore, the antiviral regimen was adjusted to dolutegravir combined with tenofovir 20.

Fourteen patients experienced immunotherapy disruption due to HBV reactivation, including 2 who discontinued therapy and 12 who delayed treatment. There were no fatal HBV reactivations. Sixteen of 20 patients achieved undetectable HBV-DNA levels after a median of 4 weeks (range, 1-14 weeks), whereas the HBV-DNA levels in 3 patients were still detectable at 4-14 weeks after HBV reactivation (183 U/mL, 599 IU/mL, and 4743 IU/mL, respectively). In the 14 patients with HBV-related hepatitis, liver enzymes returned to normal after a median of 4.5 weeks (range, 2-11 weeks).

### Efficacy

The safety of ICIs has been the major concern in most studies of ICIs in patients with advanced tumours and HBV infection, and data on the efficacy are conflicting. Among the 146 patients with known responses to ICI therapy, 14 of 88 patients with HCC (15.91%), 6 of 45 patients with NSCLC (13.33%), and 3 of 13 patients with melanoma (23.08%) had a therapeutic response (**Table 6**). Two prospective clinical studies reported details about the objective response rate (ORR) of ICIs in patients with advanced HCC. In the CheckMate 040 study, objective responses included one complete response (CR) and eight partial responses (PRs), for an ORR of 13.6%. Disease control was observed in 48 patients 4. The KEYNOTE-224 study reported five patients who achieved PR (ORR = 23.8%) 5.

Of the 10 retrospective studies included in this review, 2 did not report the efficacy of ICIs 25,28, and 7 studies, limited by sample size and experimental design, assessed the best efficacy of patients in terms of number of cases. Only one study used propensity score matching (PSM) to compare the effects of ICI treatment in patients with and without HBV infection (HBV group, n = 15; non-HBV group, n = 24). There were no statistically significant differences in the ORR (55.6% and 36.8%, P = 0.35) or overall survival (OS) (P = 0.15) between the two groups. Of the eight case reports included in the review, three only recorded HBV reactivation and the corresponding treatment. The other cases indicated best responses of CR (n = 3), SD (n = 1), and progressive disease (PD) (n = 1) 33.

Only 3 of 12 patients in the FAERS database who experienced HBV reactivation showed responses to immunotherapy, including 1 patient with NSCLC who achieved PR to pembrolizumab, 1 patient with small cell lung cancer with PD, and 1 patient with HCC with PD.

## Discussion

We screened all the published cases and meeting abstracts involving patients with advanced cancer and HBV infection receiving ICIs and identified a total of 22 publications including 633 patients. Of these, 26 (4.1%) developed HBV reactivation, including 6 cases among 79 patients in two meeting abstracts. Moreover, grade 3 or higher elevation of liver aminotransferases occurred frequently in those patients, consistent with the findings of most retrospective studies. Through the FAERS database search, we identified 12 patients with advanced cancer with HBV infections who received ICIs and developed HBV reactivation. Four of these patients died, and the data were rather limited with regard to HBV-DNA load and antiviral treatments. Hence, we should consider that HBV reactivation may not have been reported among these patients, and selective publication may underreport the risk of HBV reactivation.

As of April 2022, to the best of our knowledge, there were only two prospective clinical trials (CheckMate 040 and KEYNOTE-224) evaluating the safety and efficacy of ICIs in HCC patients with HBV infection, both of which highlighted that patients with HBV infection should receive antiviral therapy and exhibit a viral load < 100 IU/mL prior to immunotherapy 4,5. In the CheckMate 040 study, 262 HCC patients were treated with nivolumab, 66 of whom were HBsAg-positive (15 recruited in the escalation phase and 51 in the expansion phase). The ORR in the HBV-infected group was 13.6%. The KEYNOTE-224 study recruited 104 patients with advanced HCC receiving sorafenib and pembrolizumab, 22 of whom had HBV infections. In this study, the ORR in the HBV-infected group was 23.8%. Intriguingly, HBV reactivation did not occur in the 88 HBsAg-positive patients in the two studies, which might be attributable to the strict enrolment conditions. However, we could not determine whether ICIs are safe for patients with HBV infection or whether there is a definite link between ICI administration and HBV reactivation.

We included 458 patients from 12 retrospective studies (including two meeting abstracts) with advanced cancer and HBV infection who received ICIs. The study by He et al. with 202 patients, which was the largest study, recorded 7 patients with HBV reactivation. The 202 patients were divided into a low HBV-DNA group (n = 94, HBV-DNA ≤500 IU/mL) and a high HBV-DNA group (n = 108, HBV-DNA >500 IU/mL) according to their baseline HBV-DNA level. However, there was no significant difference in the incidence of HBV-associated hepatitis (*P* = 0.56) or PD-1 inhibitor disruption (*P* = 0.82) between the two groups. Further, six patients who developed HBV reactivation were treated with hepatic arterial infusion chemotherapy (HAIC). Therefore, the authors divided patients into HAIC and non-HAIC groups and found that the HBV reactivation rate in the HAIC group was higher than that in the non-HAIC group (*P* = 0.04) 32. The study by Zhang et al., which had the second largest sample size (n = 114), reported six patients with HBV reactivation, five of whom were not administered prophylactic antiviral treatment. They noted that the most significant risk factor for viral reactivation was lack of antiviral prophylaxis before immunotherapy (odds ratio [OR], 17.50 [95% CI, 1.95-157.07]; *P* = 0.004) 28. The study by Ng et al. with 62 patients indicated that 5 of 6 patients with HBV reactivation were administered antiviral drugs before ICI therapy, which differs from the results reported by Zhang et al. 8. Although their study was presented in the form of a meeting abstract lacking baseline information and treatment regimens, the authors noted that baseline HBV-DNA levels higher than 100 IU/mL were not associated with an increased risk of HBV reactivation, which was similar to the findings of Byeon et al. and He et al. 31,32. The study by Byeon et al. included 16 patients with previous HBV infections and 16 with chronic HBV infections, and they showed that patients with chronic infections were more likely to develop HBV reactivation after ICI treatment. However, there was no HBV reactivation reported in seven of the studies included in this review. There was only one study that directly examined the impact of HBV infection on the outcome of patients with advanced cancer treated with PD-1 inhibitors. The researchers evaluated the ORR, disease control rate, OS, and progression-free survival in the HBV (n = 15) and non-HBV groups (n = 24) after PSM. They found that HBV infection status did not impact the therapeutic response (the ORR) or prognosis 33. However, research about HBV reactivation caused by ICI administration among patients with advanced cancer mainly comes from retrospective studies, which provide low-level evidence and are subject to selection bias. Therefore, prospective clinical trials with HBV reactivation as the primary endpoint are needed.

We included eight case reports, of which four described patients seropositive for hepatitis B surface antigen with HBV reactivation. Three patients with NSCLC received PD-1 inhibitor and one with melanoma was treated with ipilimumab and nivolumab. Additionally, CTCAE grade 3-4 hepatitis and HBV reactivation occurred in four patients. Four of the patients did not experience HBV reactivation, three of whom were treated with prophylactic antiviral medication (entecavir) and had detectable HBV-DNA prior to ICI therapy 16,21-23. The remaining patient had malignant melanoma, and tenofovir was administered for prophylactic antiviral therapy despite the baseline HBV-DNA being undetectable 16. Although HBV reactivation in patients with advanced cancers receiving ICIs has been recorded, the lack of long-term observation data regarding ICI therapy efficacy is a limitation.

We observed three patterns of HBV reactivation in the studies included in this systematic review. The first occurred in patients with undetectable HBV-DNA and no antiviral prophylaxis before immunotherapy. In these patients, there were significant increases in HBV-DNA load and AST/ALT at virus reactivation after receiving immunotherapy. AST/ALT and HBV-DNA load returned to normal after delaying immunotherapy and administration of antiviral treatment. The second pattern involved HBV reactivation despite antiviral prophylaxis and undetectable HBV-DNA, and antiviral therapy was adjusted to control the viral infection in these patients. The third included patients undergoing antiviral prophylaxis in whom HBV-DNA was detectable. Although HBV was reactivated in these patients, AST/ALT and HBV-DNA returned to normal after antiviral treatment.

An interesting retrospective pharmacovigilance study from FAERS reported at the 2020 ASCO meeting examined HBV reactivation in patients treated with pembrolizumab, atezolizumab, or nivolumab between 2016 and 2019. This study included 15 cases of HBV reactivation related to the use of PD-1/PD-L1 inhibitors (ROR 1.2 [95% CI 0.72-1.99]). Intriguingly, only pembrolizumab was significantly correlated with HBV reactivation (ROR 2.93 [95% CI 1.57-5.46]). Unfortunately, the diagnostic criteria, treatment, and prognosis associated with HBV reactivation were not reported.

We also searched the clinical trial network, and five registered, single-arm clinical trials were identified **(Table 7)**. However, only one study's primary endpoint was HBV reactivation, whereas the remaining studies assessed the efficacy of ICIs. Because there are few prospective clinical studies on whether ICIs cause HBV reactivation, the answer to this question may mainly come from retrospective analysis.

### The mechanisms behind HBV reactivation

HBV reactivation, a serious complication in patients with cancer or receiving organ transplantation, is defined as the emergent reappearance or surge of HBV-DNA by at least 100-fold in the serum of individuals with resolved or inactive chronic HBV infection. Reactivation occurs in response to cytotoxic chemotherapeutics (e.g. vincristine), biological agents (e.g. anti-T and B lymphocyte monoclonal antibodies), or immunosuppressive drugs (e.g. glucocorticoids) 34-36. Immunosuppressive therapy can make a patient susceptible to HBV reactivation; in particular, novel agents that modulate the immune system, such as ICIs, may induce HBV reactivation. HBV reactivation has been reported to occur in 4.1% of patients with advanced cancer receiving ICIs, which raises concerns about the safety of ICIs in patients ever exposed to HBV and the risk/benefit ratio of these newly developed therapeutic modalities 28,35,37,38. Additionally, the PD-1/PD-L1 axis is a crucial suppressor of HBV-specific CD8+ T cell activities; therefore, PD-1/PD-L1 blockade could partially recover effective HBV-specific T-cell responses against viral proteins 39-42. Therefore, HBV reactivation in this circumstance appears to be a paradoxical event with no rational explanation.

**Fig 3:**
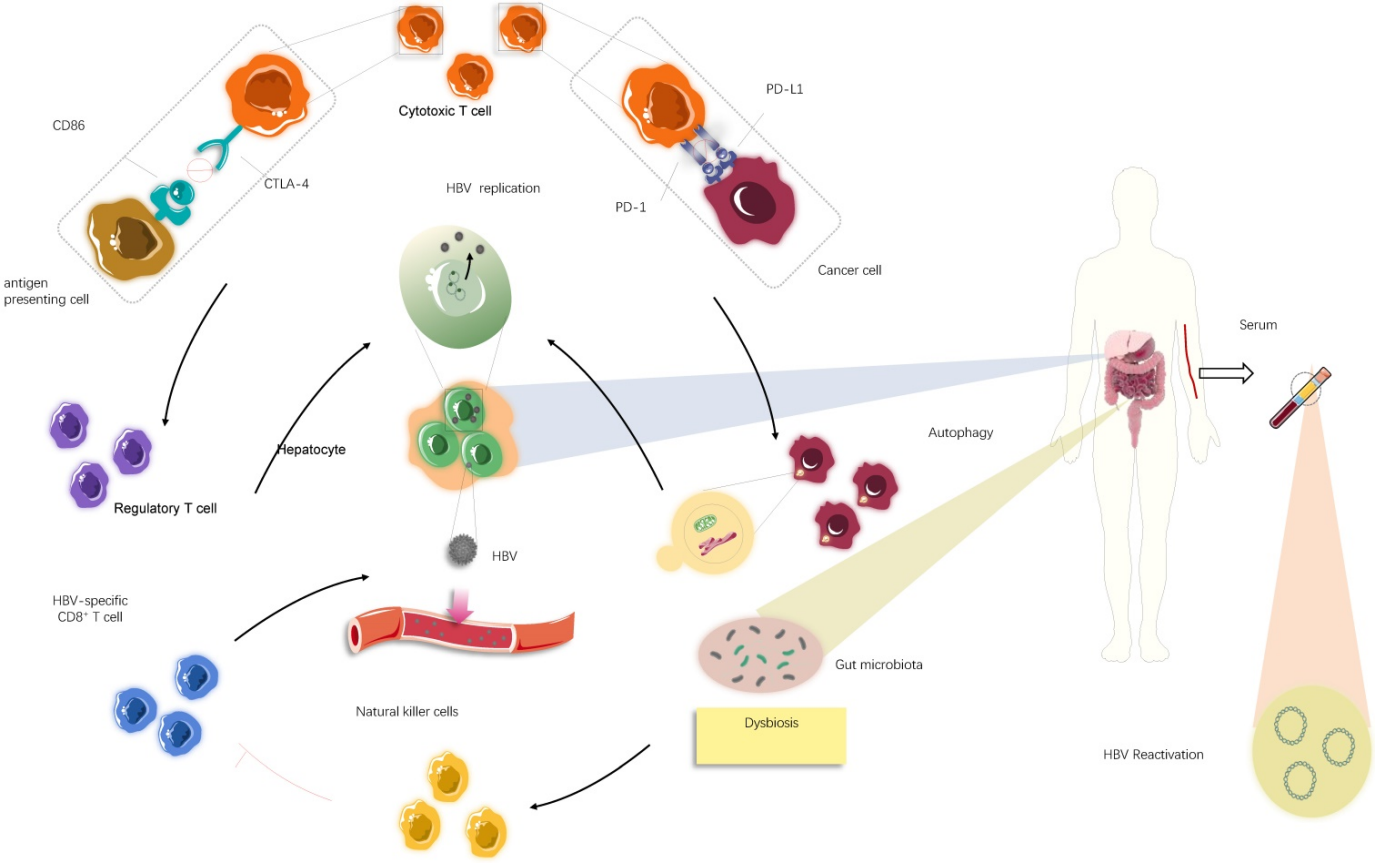
** The underlying mechanism causing HBV reactivation induced by immune checkpoint inhibitors (ICIs).** Overall, HBV reactivation has been reported to occur in approximately 4.1% of patients with advanced tumour undergoing ICIs therapies (i.e., anti-PD-1/PD-L1 and anti-CTLA-4 therapies), manifested by the re-detected HBV DNA levels in serum samples. In individuals ever infected with HBV, blockade of PD-1/PD-L1 axis could stimulate autophagic activities of cancer cells, which might be involved in further HBV replication. Apart from that, suppression of CTLA-4 may lead to increase in the frequency of regulatory T cells, thereby creating a context for HBV survival and expansion. In addition to these immediate impacts of immunological systems on HBV fate, most likely dysbiosis of gut microbiota, partly mediated by ICIs treatments, serves an immunological role by activating natural killer cells and in turn driving the exhaustion of HBV-specific T cells, consequently facilitating the release of latent HBV from damaged hepatocyte into circulation.

Although there is little information regarding the mechanism behind HBV reactivation during ICI treatment, examining virology, microbiology, and immunology in HBV carriers with advanced cancer may provide some answers (**Fig. 3**). Most importantly, it is difficult to thoroughly eradicate HBV to obtain long-lasting survival once infection occurs 36, and HBV persistence could be established by modifying host immune responses and sustained by low viral loads and dysbiosis of gut microbiota. Further, the presence of maternal hepatitis Be antigen (HBeAg) 42-45 might explain potential HBV reactivation among patients with a history of HBV infection to some extent. The innate immune system is unable to eliminate all infected hepatocytes harbouring low-level replicating HBV and/or persistent CCC HBV-DNA, which allows for a repository for HBV reactivation 28,46,47. The PD-1/PD-L1 axis also plays a critical role in maintaining immune homeostasis. PD-1 protects the body from liver damage triggered by overactive immune response to infection 28,48-53. Therefore, blocking PD-1/PD-L1 signalling might cause dysbiosis of immunoregulation, leading to the destruction of hepatocytes harbouring remaining HBV and the subsequent release of latent virus into circulation 54,55 (**Fig. 3**). Conversely, PD-1 expression is inversely correlated with proliferation and suppression capacity of regulatory T cells (Tregs) that help establish the equilibrium between immune pathology and protection, which suggests that clinical PD-1 blockade might partially impair protective immune responses, accompanied by elevated immunosuppression, eventually increasing the likelihood of HBV reactivation 55-57 (**Fig. 3**). Consistent with this idea, a recent study suggested that the frequency of Tregs is increased and levels of CTLA-4 are decreased in patients lacking CTLA-4, indicating that inhibition of CTLA-4, a crucial regulator responsible for immune homeostasis maladjustment, could promote HBV survival and expansion to some degree 58. Moreover, other immune checkpoints, such as TIGIT, LAG-3, and TIM-3, can boost tumour immune evasion and motivate the exhaustion of virus-specific T cells. The resultant immunosuppressive effects may help reactivate HBV under certain conditions in a small cohort of patients after PD-1/PD-L1 inhibitor monotherapy or alternative single application of ICIs 7. Given these questions, it is difficult to determine whether ICIs are beneficial or harmful due to a wide spectrum of potential relationships between immunotherapy, the immune system, and residual virus.

Aside from the direct effect of the immune system on virus reactivation, there may be other factors associated with the immune response affecting HBV reactivation. HBV reactivation is associated with gut microbiota, and alteration of bacterial microbiota profiles could affect the liver to determine viral fate through the gut-liver axis by several immunological signalling pathways 44,48,59,60. Certain routine therapies cause perturbations to the gut microbiota, and immunotherapy is no exception 61-64. Anti-CTLA-4 therapy can modulate the composition of gut-derived microbiota owing to the interplay between the immune response and intestinal microbiota 62. In particular, a study using a mouse model deficient for PD-1 indicated a reduction of symbiotic flora, such as *Bifidobacterium* and *Bacteroidaceae,* and an increase in pathogenic flora, such as *Enterobacteriaceae,* in the colons of PD-1^-/-^ mice compared with those of wild-type mice 65. Further, there is ample evidence indicating that increased pathogenic intestinal bacteria can facilitate liver injury mediated by excessive immune responses owing to natural killer (NK) cell activation. This may allow HBV to escape from damaged hepatocytes and enter a reactive, replicating state in individuals infected with HBV 66,67 (**Fig. 3**). In addition, the activated NK cells promote the exhaustion of virus-specific T cells to limit antiviral responses, which could promote HBV activation 68,69. Therefore, ICIs may promote considerable changes within the gut microbiota or the expansion of pathogenic bacteria, which in turn conduces construction of a distinct, supportive niche where HBV reactivation occurs. Despite the effects of immune factors on HBV reactivation, several factors independent of host immunity are implicated in this process, such as interconnected alterations in major cellular signalling and behaviours of the infected cells 70.

Emerging evidence indicates autophagy mediated by Akt/mTOR signalling occurs during HBV replication since HBV can hijack components of the autophagic pathway 71-73. A study on the intrinsic functions of PD-1/PD-L1 suggested that PD-L1 blockade may cause PD-L1 attenuation and subsequently augment autophagy because of mTORC1 activation driven by tumour-expressed PD-L1 74. Therefore, it is plausible that autophagy enhanced by anti-PD-1/PD-L1 immunotherapy, if it occurs, will potentiate viral replication, which would lead to detectable serum HBV-DNA. In addition to the involvement of autophagy in stimulating HBV replication, inhibition of cell proliferation following cytotoxic chemotherapy appears to be advantageous for viral replication, with intracellular organelles and nutrients preferentially serving the virus rather than the cell 67. Related to this observation, PD-L1 antibody therapy exhibits adverse impacts on tumour cell growth and remarkably reduces cell proliferation, which could explain why HBV reactivation can take place after ICI treatment 74. Although counterintuitive, two therapies affecting immune functions in opposing directions may converge to contribute to the severe outcome represented by HBV reactivation in very similar manners.

### Clinical implications

Because HBV reactivation in the context of ICIs remains unsolved, for patients with cancer and HBV infection receiving ICIs, we recommend regular monitoring of the HBV-DNA level, examination of serological characteristics, and antiviral prophylaxis, along with the optimization of monitoring, prevention, and management of HBV reactivation throughout the course of treatment. Based on the studies discussed above, we further suggest that all patients should be screened for HBV using HBsAg and HBcAb. Additionally, HBV-DNA testing is recommended in areas with a high incidence of hepatitis B. If the test result indicates a chronic HBV infection, initiation of prophylactic antiviral treatment is recommended, although it is still unclear how long prophylactic treatment should be continued. With regard to those with past HBV infection, regular monitoring of serum ALT and HBV-DNA is suggested. If HBV-positive patients display elevated levels of serum liver transaminases during ICI treatment, HBV-DNA testing should be considered. In addition to monitoring the levels of liver aminotransferases, it is critical to differentially diagnose whether the elevated aminotransferase is due to liver damage from hepatitis B reactivation or immune liver damage.

### Strengths and limitations

This review comprehensively explores current studies examining the reactivation of HBV caused by ICIs. It is the first study to summarize important clinical information of 20 patients with advanced cancer and HBV infection who developed HBV reactivation after ICI treatment (Table 5). We also outline three patterns of HBV reactivation. In addition, we used the FAERS database to include previously unreported patient information. Because there are inconsistencies between basic research and clinical reports, we also examined the mechanism of HBV reactivation, including mechanisms associated with intestinal microorganisms, viruses, and the immune system.

There were several limitations to our systematic review. First, the data were mainly obtained from retrospective studies (n = 10, 45.5%) and case reports (n = 8, 36.4%), with two prospective clinical trials (9.1%) and two meeting abstracts (9.1%). Inevitably, the data extracted from studies may lead to selection bias and heterogeneity in treatment response and adverse events. Even the representativeness of prospective studies may be affected by strict inclusion criteria. Second, there were few published cases, and those detailing HBV reactivation were not comprehensive. For instance, the cases were mainly distributed in East Asia, which may restrict the application of our findings to other regions. Finally, the LAG-3 immune checkpoint inhibitor relatlimab was approved by the FDA on March 18, 2022, and currently, there are an unprecedented number of clinical trials of novel immune checkpoint inhibitors. (The data on ongoing clinical trials of relatlimab and TIM3 ICIs are detailed in Table S1 and Table S2). However, limited by the current stage of research, there are no reports of HBV reactivation caused by these novel immune checkpoint inhibitors. Future studies will be important for understanding the relationship between HBV reactivation and other ICIs.

## Conclusion

HBV reactivation might occur in HBV-positive cancer patients receiving ICI therapy. As HBV reactivation can be effectively controlled with current antiviral drugs, ICIs seem to be a safe and effective treatment option in this patient population. However, there is still a need for clinical monitoring of liver enzymes and HBV-DNA levels. Ongoing prospective clinical trials will shed further light on the safety and efficacy of ICI therapy in cancer patients with HBV infections.

## Supplementary Material

Supplementary tables.

## Figures and Tables

**Table 1:** Select Studies of Immune Checkpoint Inhibitor Therapy in Patients with HBV Infection and Advanced Cancer

Reference	Year	Sample Size	Tumor Type	Number of HBV Infection	ICIs Therapy	Viral Load^*^	Antiviral Prophylaxis		Antiviral Therapy	HBV Reactivation Number of Events	Adverse Events	ORR (%)
**Prospective clinical trails (n=2)**												
El-Khoueiry AB	2017	226(66HBV)	HCCs	15 in escalation phase/51 in expansion phase	Nivolumab	Undetectable (66)	Yes (All patients were required to be receiving effective antiviral therapy)		Nil (0/66)	(0/66) No patient had reactivation of HBV, no anti- HBs sero conversion	in the dose-expansion phase, HBV infected group Diarrhoea(G3/4):1(2%)	CR(1) PR(8) SD(39) PD(17) NR(1) 13.6%
Zhu AX	2018	104(22HBV)	HCCs	Hepatitis B positive (22)	Pembrolizumab	Undetectable (22)	Yes (All patients were required to be receiving effective antiviral therapy)		Nil (0/22)	(0/22) No cases of flares of hepatitis B virus occurred.	Not specifically reported for HBV positive patients.	PR(5) SD+PD(16) NR(1) 23.8%
**Retrospective case series (n=10)**												
Ravi S	2014	9(5HBV)	Melanoma (9)	5 HBV positive (active/inactive 3/2)	Ipilimumab (9)	Undetectable/Detected (2/3)	Entecavir (2) Tenofovir(2) Nil (1)	Nil (0)		(0/5) No patient had reactivation of HBV, (3/5) HBV-DNA levels remained undetectable	Only ALT elevation reported. ALT elevation G1: 2	SD(1) PD(4)
Wen X	2016	23(11HBV)	Melanoma (11)	11 pre-existing hepatitis B virus (HBV) infection	Ipilimumab (3) Pembrolizumab (5) Concurrent Ipilimumab -pembrolizumab (3)	Undetectable/Detected (10/1)	Entecavir (3) Nil (8)	Nil (0)		(0/11) No patient had reactivation of HBV, (6/11) HBV-DNA levels remained undetectable	Any G: (8/11)73%, ALT/AST increaseG3:(1/11)	NR
Kothapalli A	2018	7(5HBV/2HCV)	NSCLC(4) Melanoma(1)	Chronic HBV (1), Possible Past HBV (1), Past HBV (3, 1 of these had HCV co-infection)	Nivolumab(4) Pembrolizumab(1)	Unknown(5)	Unknown(5)	Nil (0/5)		(0/14) No patient had reactivation of HBV	ALT rise G1:(3), Nil(2)	PR(1) SD(2) PD(2)
Tio M	2018	46(14HBV/14HCV/12HIV/6Organ transplant)	Melanoma (9) Mesothelioma (1)Glioblastoma (1) HCC (1)Gastric (1)Urothelial (1)	hepatitis B infection(14)	Pembrolizumab (9)Nivolumab (4)Sequentialpembrolizumab + Ipilimumab (1)	Undetectable/Detected (4/4) Unknown (6)	Tenofovir (3) Entecavir (5) Nil (6)	Nil (0/14)		(0/14) No patient had reactivation of HBV	hypothyroidism G2:(1), rashG1:(1) G2:(1) pneumonitis G2:(1) vitiligo G1:(1)	CR(1)/PR(2) /SD(8) (21.4%).
Zhang X	2019	114	NSCLC (13) HCC (28) Melanoma (14) Nasopharyngeal carcinoma (35) Lymohoma(8) Others(16)	HBsAg positive (114)	Anti-PD-1/PDL-1 monotherapy (83) Combination therapy (31) Immunotherapy monotherapy(83): pembrolizumab, nivolumab, toripalimab, camrelizumab, sintilimab, atezolizumab. Combinations of immunotherapy and chemotherapy (22), targeted agent ((osimertinib [n = 1], bevacizumab [n = 1],regorafenib [n = 1], apatinib[n = 1], sunitinib [n = 1], nimotuzumab [n = 2], cetuximab [n = 1]) and ipilimumab (n = 2)	Undetectable/Detectable (79/35)	Tenofovir (5) Entecavir (68) Lamivudine (10) Telbivudine (1)Adefovir (1) Nil (29)	Entecavir (4) Entecavir + Tenofovir (1)Nil (1)		(6/114)These 6 patients had baseline HBV- DNA negative, only 1 patients receiving prophylactic entecavir	hepatitis G1-2: (35) G3-4: (10)	NR
Shah NJ	2019	34 (16 HBV/ 18 HCV	HCC (16) NSCLC (10) RCC (3)Gastric (1) SCLC (1)H&N (1)Anal SCC (2)	16 HBV chronic viral infections (1 of these had HCV and 1 of these had HIV co-infection. 8 patients had positive HBsAg, 4 patients were HBsAg (-), HBsAb (-), and HBcAb (+), and 3 patients were HBsAg (-), HBsAb (+), and HBcAb (+). One patient's HBV status was unknown.)	Anti-PD-(L)1 monotherapy (30) Anti-PD-(L)1 plus chemotherapy (3)Concurrent Ipilimumab -Nivolumab (1)	Undetectable/Detectable (8/5) Unknown (3)	Tenofovir (6) Entecavir (3) Nil (7)	Nil(0)		No patient had reactivation of HBV	Any grade 15 (44%)Grade≥ 3: 10 (29%)	6 PR (18%)
Pertejo-Fernandez A	2020	19(16HBV/3HCV)	NSCLC	HBV (16, 2 of these had HCV co-infection)	Anti-PD-1/PDL-1 monotherapy(14) Combination therapy(2)Immunotherapy monotherapy(14):nivolumab(3), pembrolizumab(7), atezolizumab(3), durvalumab(1)Combinations of immunotherapy and chemotherapy(2):pembrolizumab+chemotherapy(1), ipilimumab+nivolumab(1)	Undetectable/Detectable (11/4), one is N/A	Tenofovir (2) Entecavir (1) Nil(13)	Nil(0)		No patient (0)	Neutropenia G4:(1) Colitis G3:(1) Diabetes mellitus G3:(1)	SD(7) PD(4) PR(5) 31%
Byeon S	2020	61(32HBV)	NSCLC	HBV (32, 16 past HBV infections and 16 chronic HBV infections)	Nivolumab(15) Pembrolizumab (17)	Undetectable/Detectable (23/4), N/A(5)	Entecavir(10), Tenofovir(1), Lamivudine(3) Nil(18)	Entecavir(2), Tenofovir(1)		(3/32) 2 patients had baseline HBV- DNA undetectable, 1 patient had an HBV DNA level of 1553 IU/mL before treatment, then rose to 11 317 IU/mL after 1 month of pembrolizumab treatment	Fatigue G2:(1) DNA seroconversion:(1)Pneumonitis G2:(1),G4:(1)Hyperglycemia G3:(1),G2(1)AST elevation G3:(6),G2:(1),G1(2) ALT elevation G4(2), G3(3),G2(1),G1(3)	SD(9) PD(11) PR(6) Not evaluate(6) 18.8%
He MK	2021	202 (202 HBV)	HCC	HBsAg positive and HBV-DNA positive (202)	Nivolumab (50) Pembrolizumab (45) Toripalimab (59) Sintilimab (35) Camrelizumab (13) Combinations of immunotherapy and HAIC (84)	HBV-DNA Low group ( ≤ 500 IU/ml N = 94) High group ( > 500 IU/ml N = 108)	Entecavir (148) Tenofovir (51) Other (3)	Entecavir(5), Tenofovir(2)		7 patients had HBV reactivation, 5 patients in the HBV-DNA low group and 2 patients in the HBV-DNA high group.	Hepatitis All grades (49), Grade 3/4 (14)	Not reported
Zhong L	2021	120(43HBV)	HCC (32) Lung cancer (2) Esophgeal cancer (1) Melanoma (4) Others (4)	HBsAg positive (43)	Anti-PD-1 monotherapy(13) Combinations of immunotherapy and chemotherapy/targeted agent (30)	Undetectable/Detectable (20/14), N/A(9)	All patients (n=43) with HBV received regular antiviral therapy	All patients (n=43) with HBV received regular antiviral therapy		No patient (0)	Not reported	CR (0) PR (5) SD(9) PD (12) 11.63%
**Case reports (n=8)**												
Sharma A	2013	2(1HBV/1HCV)	Melanoma	1 HBeAb(+)	Ipilimumab	Undetectable	tenofovir		tenofovir	0	Not reported	PD
Koksal AS	2017	1	Melanoma	1 HBsAg(+)	Ipilimumab+Nivolumab	Unknown	No		tenofovir disoproxil fumarate	1	hepatitis (G3-4)	Not reported
Pandey A	2018	1	NSCLC (ADC)	1 HBsAg(+)	Pembrolizumab	Unknown	No		tenofovir disoproxil fumarate	1	hepatitis (G3-4)	Not reported
Ragunathan K	2017	1	NSCLC (ADC)	1 HBsAg(+)	Pembrolizumab	Unknown	No		Unknown	1	hepatitis (G3-4)	Not reported
Lake AC	2017	1	NSCLC (ADC)	1 HBsAg(+) HBeAg (+) /HIV	Nivolumab	Undetectable	No		tenofovir disoproxil fumarate	1	hepatitis (G3-4)	CR
Liu Z	2019	1	HCC	Chronic HBV infection	Pembrolizumab	Detectable	Entecavir		Entecavir	0	Diarrhea G3, Thrombocytopenia G4 occurred after the administration of Lenvatinib	CR
Akar E	2019	1	RCC(clear cell)	HBsAg(+), also HDV-DNA(+)	Nivolumab	Detectable	Entecavir		Entecavir	0	Nil	SD
Duan X	2020	1	HCC	Chronic HBV infection	Sintilimab	Detectable	Entecavir		Entecavir	0	fatigue G1, thrombocytopenia G1, AST elevation G1, ALT elevation G2	CR
**ASCO meeting abstract&Poster (n=2)**												
Kennedy Ng	2020	114(62HBV/13HCV/)	HCCs	62 HBV positive	Most received ICI as monotherapy (62%),most commonly a PD-1(58.8%)	Undetectable/Detectable (15/40)	Unknown (Most HBV patients (57, 91.9%) were on antiviral therapy)		Unknown	6 /62 (1 patient were on baseline anti-virals before ICI initiation, 5 were not. 1 required treatment discontinuation. )	Any Grades(79 69.3%), Grade3 or 4(17, 14.9%)	PR(21, 18.4%) SD(37, 32.5%) PD(46, 40.4%) ORR(21, 18.4%)
Chongrui Xu	2020	17	NSCLC	17 HBsAg(+)	Nivolumab(4) Pembrolizumab(5) Nivolumab+chemo(2) Pembrolizumab+chemo(5)	Unknown	8 patients received anti-virus therapy including entecavir and adfovir during the treatment		Nil	0	Transaminase or bilirubin elevation(G1):6, Transaminase elevation(G3):1, bilirubin elevation(G4):1	NR
												

HBV=Hepatitis B virus, HCV=Hepatitis C virus, HIV=Human immunodeficiency virus; HCC=Hepatocellular Carcinoma; NSCLC=Non-Small cell lung cancer; ADC=adenocarcinoma; SCLC=Small cell lung cancer; H&N=Head and neck squamous cell carcinoma,RCC=Renal cell carcinoma; CR= complete response, PR=partial response, SD=stable disease, PD=progressive diseas; NR=Not Report;Nil=Zero; ALT=Alanine transaminase; AST=Aspartate aminotransferase; HBsAg=Hepatitis B surface antigen, HBsAb=Hepatitis B surface antibody, HBcAb=Hepatitis B core antibody, HBeAg=Hepatitis B e antigen, HBeAb=Hepatitis B e antibody HAIC=Hepatic arterial infusion chemotherapy. *Viral Load (IU/ml) Detectable HBV-DNA≥100 IU/ml;Undetectable<100.

**Table 2:** Characteristics of 538 patients with HBV infection and Advanced-Stage Cancer included in Review

Characteristic	No	%
Age, mean(range)	58.5(16-88)	
Sex		
Male	385	72%
Female	103	19%
Unknown	50	9%
Disease type (n=538)		
NSCLC	70	13.0%
Melanoma	46	8.6%
HCC	353	65.6%
Lymphoma	9	1.7%
NPC	35	6.5%
Other	25	4.6%
ICI treatment (n=538)		
Nivolumab	144	80.4%
Pembrolizumab	110	61.5%
Ipilimumab	9	5.0%
Pembrolizumab+Ipilimumab	4	2.2%
Atezolizumab	3	1.7%
Durvalumab	1	0.6%
Ipilimumab+Nivolumab	2	1.1%
Sintilimab	36	20.1%
Camrelizumab	13	7.3%
Toripalimab	59	33.0%
Anti-PD-1/PDL-1(not specified)	114	21.2%
Anti-PD-1 (not specified)	43	8.05
Baseline viral load recorded (n=444)		
Undetectable	237	53.4%
Detectable	176	39.6%
Unknown	31	7.0%
Antiviral prophylaxis (n=416)		
Entecavir	240	57.7%
Tenofovir	65	15.6%
Lamivudine	13	3.1%
Telbivudine	1	0.2%
Adefovir	1	0.2%
Unknown	96	23.1%

**Table 4:** Adverse Events Reported in The Literature for Patients with Advanced Cancer and Hepatitis B Infection Treated with ICI

	Any grade	Grade1	Grade2	Grade3-4
**Treatment-related Aes**				
Diarrhoea	1	0	0	1
Rash	2	1	1	0
Fatigue	2	1	1	0
Hypothyroidism	1	0	1	0
Pneumonitis	3	0	2	1
Vitiligo	1	1	0	0
Colitis	1	0	0	1
**Laboratory-treatment related Aes**				
ALT elevation	122	49	37	36
AST elevation	63	44	1	18
Hyperglycemia	4	0	1	3
Thrombocytopenia	2	1	0	1
Neutropenia	1	0	0	1
**Unspecified Aes**	27	0	0	27

ALT=Alanine transaminase; AST=Aspartate aminotransferase

**Table 3:** Characteristics of 12 patients with HBV infection and Advanced-Stage Cancer included in the FDA Adverse Event Reporting System

Patient	Age	Gender	Country	Cancer Type	ICIs	Combined treatment	Outcome	Received Date	Response
1	71	M	USA	Malignant Melanoma Stage Iv	Pembrolizumab	No	Other	20-Apr-19	-
2	Not Specified	F	USA	Neoplasm Malignant	Pembrolizumab	No	Other	7-Jun-17	-
3	36	F	France	Non-Hodgkin'S Lymphoma	Pembrolizumab	Adcetris	Other	11-Sep-18	-
4	57	M	Bulgaria	Non-Small Cell Lung Cancer Metastatic	Pembrolizumab	Methylprednisolone	Died	22-Apr-19	PR
5	Not Specified	M	Bulgaria	Small Cell Lung Cancer	Pembrolizumab	No	Other	26-Feb-19	PD
6	Not Specified	Not Specified	USA	-	Pembrolizumab	No	Other	22-Mar-16	-
7	45	M	Korea	Hepatocellular Carcinoma	Nivolumab	No	Died	19-Jan-18	PD
8	Not Specified	Not Specified	Japan	Malignant Melanoma	Nivolumab	No	Other	14-Dec-15	-
9	Not Specified	Not Specified	USA	Hodgkin'S Lymphoma	Nivolumab	Rituximab etc	Died	23-Jan-19	-
10	Not Specified	M	HK	Transitional Cell Carcinoma	Atezolizumab	No	Other	30-Mar-18	-
11	83	F	Belgium	Triple Negative Breast Cancer	Atezolizumab	Paclitaxel	Other	15-Jun-20	-
12	67	M	France	Lung Adenocarcinoma	Durvalumab	No	Died	18-Nov-19	-

**Table 5:** The Situation of 13 Patients with Advanced Cancer Who Had Hepatitis B Reactivation After Using ICIs

	Patients Characteristics				Baseline					At reactivation				Antiviral Treatment	Time for Achieving HBV-DNA Undetectable (Weeks)	Time for ALT Recovery (Weeks)
Reference	Patient	Age	Gender	Country	Cancer Type	ICIs	Combined Treatment	HBV-DNA	Antiviral Prophylaxis	Weeks From Start of ICI	HBV-DNA(copies/ml)	Peak AST/ALT(U/L)	Anti-PD-1/PD-L1 Therapy Disruption			
Zhang X 2019(a)	1	48	M	China	NPC	Camrelizumab	No	Undetectable	No	3	7.81×103	ALT=191.4	Delayed	Entecavir	1	2
	2	47	M	China	NPC	Camrelizumab	No	Undetectable	No	16	6.98 × 104	ALT=203	Delayed	Entecavir	4	4
	3	39	M	China	Melanoma	Pembrolizumab	No	Undetectable	No	28	2.10 × 103	ALT=27.6	No	Not use	5	Not reported
	4	36	M	China	HCC	Nivolumab	No	Undetectable	Entecavir	12	1.80 × 103	ALT=298	Discontinued	Entecavir+tenofovir	1	3
	5	45	M	China	H&NSCC	Toripalimab	No	Undetectable	No	35	4.04 × 106	ALT=281.2	Delayed	Entecavir	3	6
	6	41	F	China	Sarcoma	Nivolumab	No	Undetectable	No	20	6.00 × 107	ALT=465.1	Not reported	Entecavir	8	4
Byeon S 2020	7	59	M	Korea	NSCLC	Nivolumab	No	70 IU/ml	Tenofovi	3	2.81× 103	AST G1	Delayed	Tenofovi	4 weeks PD die	Not reported
	8	60	M	Korea	NSCLC(adenocarcinoma)	Pembrolizumab	No	Undetectable	Entecavir	4	1.48 × 103	Normal	Delayed	Entecavir	4	Not reported
	9	45	M	Korea	NSCLC(adenocarcinoma)	Pembrolizumab	No	1553 IU/ml	Entecavir	4	1.13 × 104	Normal	No	Entecavir	599 IU/ml	Not reported
Koksal AS 2017	10	56	M	Turkey	Melanoma	Ipilimumab*4 cycle,Nivolumab	No	Unknown	No	14	2.45 × 105	845/888	No	Tenofovir	11week(183IU/ml)	11
Pandey A 2018	11	51	M	USA	NSCLC(adenocarcinoma)	Pembrolizumab	Whole Brain radiation	Unknown	No	4	>1.70× 108	670/994	No	Tenofovir	10	10
Ragunathan K 2017	12	51	M	USA	metastatic adenocarcinoma of the lung	Pembrolizumab	No	Unknown	No	2	>170.0 IU/ml	217/615	Not reported	Not reported	Not reported	Not reported
Lake AC 2017	13	72	M	USA	NSCLC(adenocarcinoma)	Nivolumab	No	Undetectable	Dolutegravir/Abacavir	13	>1.7 × 105	520/474	Delayed	Dolutegravir /Tenofovir	14	9
He MK 2021	14	Not reported	M	China	HCC	Pembrolizumab	No	1.65e2 IU/mL	Tenofovir	6	2.85×104	ALT=198.2	Discontinued	Entecavir	7	5
	15	Not reported	M	China	HCC	Pembrolizumab	HAIC	3.83e6 IU/ml	Tenofovir	14	4.86×103	ALT=271.6	Delayed	Tenofovir	9	6
	16	Not reported	M	China	HCC	Toripalimab	HAIC	Undetectable	Entecavir	6	1.31×103	ALT=39.6	No	Entecavir	1	Not reported
	17	Not reported	M	China	HCC	Nivolumab	HAIC	5.86e3IU/ml	Entecavir	12	1.58×103	ALT=208.5	Delayed	Entecavir	3	3
	18	Not reported	M	China	HCC	Sintilimab	HAIC	Undetectable	Entecavir	4	2.98×103	ALT=151	Delayed	Entecavir	4	3
	19	Not reported	M	China	HCC	Toripalimab	HAIC	Undetectable	Entecavir	16	1.21×103	ALT=174.8	Delayed	Tenofovir	2	3
	20	Not reported	M	China	HCC	Toripalimab	HAIC	Undetectable	Entecavir	4	1.21×103	ALT=378.8	Delayed	Entecavir	7	6

**Table 6:** Objective Response Rates Reported in The Literature for Patients with Advanced Cancer and Hepatitis B Infection Treated with ICI

		Response(No. of Patients)				ORR
Tunor	Number of Patients With Known Response	CR	PR	SD	PD	
HCC	88	6	8	50	24	15.91%
NSCLC	45	0	6	18	16	13.33%
Melanoma	13	1	2	4	6	23.08%

CR= complete response, PR=partial response, SD=stable disease, PD=progressive diseas; ORR=Objective response rate.

**Table 7:** Ongoing Clinical Trials of ICIs in Patients With HBV Infection and Advanced-Stage Cancer

Trial	NCT Trial No	Location	ICI	Tumor	Phase	Study Type	Sample size	Primary Endpoint
P1101 and Anti-PD1 for After Curative Surgery of Hepatitis B-related Hepatocellular Carcinoma	NCT04233840	Taiwan	Nivolumab	HCC	I/II	Interventional (Clinical Trial)	72	Dose-limiting Toxicity/Recurrence-free survival
Pembrolizumab in Hepatocellular Carcinoma	NCT03419481	Hongkong	pembrolizumab	HCC	II	Interventional (Clinical Trial)	30	Response rate
Atezolizumab Plus Bevacizumab for Patients With Advanced Hepatocellular Carcinoma (HCC) and Chronic Hepatitis B Virus (HBV) Infection	NCT04180072	Taiwan	Atezolizumab	HCC	II	Interventional (Clinical Trial)	48	Best overall response rate Best overall response rate
Durvalumab for Advanced Hepatocellular Carcinoma in Patients With Active Chronic Hepatitis B Virus Infection	NCT04294498	Taiwan	Durvalumab	HCC	II	Interventional (Clinical Trial)	43	The rate of HBV reactivation
Safety and Immunotherapeutic Activity of Cemiplimab in Participants With HBV on Suppressive Antiviral Therapy	NCT04046107	Los Angeles	Cemiplimab	Nil	I/II	Interventional (Clinical Trial)	30	Targeted safety events/Number of discontinue treatment

HCC=Hepatocellular Carcinoma; HBsAg=Hepatitis B surface antigen.

## Abbreviations

ALT: alanine aminotransferase
ASCO: American Society of Clinical Oncology
AST: aspartate aminotransferase
CI: confidence interval
CR: complete response
CTCAE: Common Terminology Criteria for Adverse Events
CTLA-4: cytotoxic T lymphocyte antigen 4
FAERS: Food and Drug Administration Adverse Event Reporting System
HAIC: hepatic arterial infusion chemotherapy
HBeAg: hepatitis B e antigen
HBsAg: hepatitis B surface antigen
HBV: hepatitis B virus
HCC: hepatocellular carcinoma
HIV: human immunodeficiency virus
ICI: immune checkpoint inhibitor
NK: natural killer
NSCLC: non-small cell lung cancer
OR: odds ratio
ORR: overall response rate
OS: overall survival
PD: progressive disease
PD-1: programmed cell death 1
PD-L1: programmed cell death ligand 1
PR: partial response
PSM: propensity score matching
ROR: reporting odds ratio
SIT: superinfection therapy
Treg: regulatory T cell
