# Supplementary material for: Hepatitis B Virus Reactivation in Cancer Patients Undergoing Immune Checkpoint Inhibitors Therapy: A Systematic Review
Source: J Cancer. 2022 Oct 31;13(14):3539–53. doi: 10.7150/jca.77247 (PMC9723987; doi:10.7150/jca.77247)
Supplement: Supplementary file 1 — Supplementary tables. [file jcav13p3539s1.pdf]

Table S1 Clinical Trials of Relatlimab (Anti LAG3)

Rank	Clinical trial	Status	Study Results	Conditions
1	NCT03470922	Active,not recruiting	Has Results	Melanoma
2	NCT03662659	Active,not recruiting	Has Results	Gastric Cancer
3	NCT02488759	Active,not recruiting	Has Results	Various Advanced Cancer
4	NCT04112498	Active,not recruiting	No Results Available	Cancer
5	NCT03044613	Active,not recruiting	No Results Available	Gastric Cancer
6	NCT03867799	Active,not recruiting	No Results Available	Metastatic Colorectal Cancer
7	NCT01968109	Active,not recruiting	No Results Available	Neoplasms by Site
8	NCT02519322	Active, not recruiting	No Results Available	Cutaneous Melanoma|
9	NCT02966548	Active,not recruiting	No Results Available	Cancer
10	NCT02996110	Active,not recruiting	No Results Available	Advanced Cancer
11	NCT02935634	Active,not recruiting	No Results Available	Advanced Gastric Cancer
12	NCT03335540	Active,not recruiting	No Results Available	Advanced Cancer
13	NCT02061761	Completed	No Results Available	Hematologic Neoplasms
14	NCT05170659	No longer available	No Results Available	
15	NCT05170685	No longer available	No Results Available	
16	NCT05498480	Not yet recruiting	No Results Available	Advanced Solid Tumors
17	NCT05418972	Not yet recruiting	No Results Available	Stage II Melanoma
18	NCT05255601	Not yet recruiting	No Results Available	Lymphoma
19	NCT05428007	Not yet recruiting	No Results Available	Melanoma
20	NCT05347212	Not yet recruiting	No Results Available	Renal Medullary Carcinoma
21	NCT04552223	Recruiting	No Results Available	Melanoma

22	NCT04658147	Recruiting	No Results Available	Hepatocellular Carcinoma
23	NCT04095208	Recruiting	No Results Available	Soft Tissue Sarcoma
24	NCT03623854	Recruiting	No Results Available	Chordoma|
25	NCT03743766	Recruiting	No Results Available	Melanoma
26	NCT04080804	Recruiting	No Results Available	Head and Neck Squamous Cell Carcinoma (HNSCC)
27	NCT04913922	Recruiting	No Results Available	Acute Myeloid Leukemia
28	NCT05002569	Recruiting	No Results Available	Melanoma
29	NCT03642067	Recruiting	No Results Available	Colorectal Adenocarcinomas
30	NCT04567615	Recruiting	No Results Available	Hepatocellular Carcinoma
31	NCT05328908	Recruiting	No Results Available	Colorectal Neoplasms
32	NCT05337137	Recruiting	No Results Available	Carcinoma, Hepatocellular
33	NCT03607890	Recruiting	No Results Available	Refractory MSI - H Solid Tumors Prior of PD-(L) 1 Therapy|
34	NCT03978611	Recruiting	No Results Available	Melanoma
35	NCT03521830	Recruiting	No Results Available	Basal Cell Carcinoma
36	NCT04326257	Recruiting	No Results Available	Squamous Cell Carcinoma of the Head and Neck
37	NCT04623775	Recruiting	No Results Available	Non-small Cell Lung Cancer
38	NCT04205552	Recruiting	No Results Available	NSCLC Stage II|NSCLC, Stage IIIA|NSCLC, Stage I
39	NCT04611126	Recruiting	No Results Available	Metastatic Ovarian Cancer
40	NCT05134948	Recruiting	No Results Available	Advanced Solid Tumors
41	NCT03459222	Recruiting	No Results Available	Advanced Cancer
42	NCT05148546	Recruiting	No Results Available	Renal Cell Carcinoma

43	NCT03610711	Recruiting	No Results Available	Gastroesophageal Cancer
44	NCT04062656	Recruiting	No Results Available	Gastric Cancer
45	NCT03310619	Recruiting	No Results Available	Lymphoma,
46	NCT02465060	Recruiting	No Results Available	Advanced Malignant Solid tumor
47	NCT04150965	Recruiting	No Results Available	Multiple Myeloma
48	NCT04866810	Recruiting	No Results Available	Melanoma
49	NCT03724968	Terminated	Has Results	Metastatic Melanoma
50	NCT02750514	Terminated	Has Results	Advanced Cancer
51	NCT03704077	Withdrawn	No Results Available	Gastric Cancer

Table S2 Ongoing Clinical Trials of Anti TIM-3 in Cancer patients

Drugs	Stage	Biologic Classification	HBV reactivation	Clinical Trial Status
sabatolimab	III	anti-TIM3 monoclonal antibody	0	recruiting
MAS825	II	anti-TIM3 monoclonal antibody	0	recruiting
cobolimab（TSR-022）	I/II	anti-PD-1 and Anti-TIM-3 Bispecific Antibody	0	recruiting

BGB-A425	I/II	anti-TIM3 monoclonal antibody	0	recruiting
BMS-986258	I/II	anti-TIM3 monoclonal antibody	0	Active, not recruiting
INCAGN2390	I/II	anti-TIM3 monoclonal antibody	0	Completed
LBL003	I	anti-TIM3 monoclonal antibody	0	recruiting
LY3321367	I	anti-TIM3 monoclonal antibody	0	Active, not recruiting
LY3415244	I	anti-PD-L1 and Anti-TIM-3 Bispecific Antibody	0	Terminated Has Results
SHR-1702	I	anti-TIM3 monoclonal antibody	0	Active, not recruiting
Sym023	I	anti-TIM3 monoclonal antibody	0	Completed Has Results
TQB2618	I	anti-TIM3 monoclonal antibody	0	Recruiting
Sym023	I	anti-TIM3 monoclonal antibody	0	Completed （Has Results）
AZD7789	I/II	PD-1/TIM-3 bispecific monoclonal antibody	0	Recruiting
